# The Left and Right Ventricles Respond Differently to Variation of Pacing Delays in Cardiac Resynchronization Therapy: A Combined Experimental- Computational Approach

**DOI:** 10.3389/fphys.2019.00017

**Published:** 2019-02-01

**Authors:** Erik Willemen, Rick Schreurs, Peter R. Huntjens, Marc Strik, Gernot Plank, Edward Vigmond, John Walmsley, Kevin Vernooy, Tammo Delhaas, Frits W. Prinzen, Joost Lumens

**Affiliations:** ^1^Cardiovascular Research Institute Maastricht (CARIM), Maastricht University, Maastricht, Netherlands; ^2^IHU-LIRYC Electrophysiology and Heart Modeling Institute, Pessac, France; ^3^Department of Cardiology, Maastricht University Medical Center, Maastricht, Netherlands; ^4^Institute of Biophysics, Medical University of Graz, Graz, Austria; ^5^Univeristy of Bordeaux, IMB UMR 5251, Talence, France

**Keywords:** cardiac resynchronization therapy, right ventricle, optimization, computer simulation, therapy optimization studies, CircAdapt, hemodynamics, dyssynchrony

## Abstract

**Introduction:** Timing of atrial, right (RV), and left ventricular (LV) stimulation in cardiac resynchronization therapy (CRT) is known to affect electrical activation and pump function of the LV. In this study, we used computer simulations, with input from animal experiments, to investigate the effect of varying pacing delays on both LV and RV electrical dyssynchrony and contractile function.

**Methods:** A pacing protocol was performed in dogs with atrioventricular block (*N* = 6), using 100 different combinations of atrial (A)-LV and A-RV pacing delays. Regional LV and RV electrical activation times were measured using 112 electrodes and LV and RV pressures were measured with catheter-tip micromanometers. Contractile response to a pacing delay was defined as relative change of the maximum rate of LV and RV pressure rise (dP/dt_max_) compared to RV pacing with an A-RV delay of 125 ms. The pacing protocol was simulated in the CircAdapt model of cardiovascular system dynamics, using the experimentally acquired electrical mapping data as input.

**Results:** Ventricular electrical activation changed with changes in the amount of LV or RV pre-excitation. The resulting changes in dP/dt_max_ differed markedly between the LV and RV. Pacing the LV 10–50 ms before the RV led to the largest increases in LV dP/dt_max_. In contrast, RV dP/dt_max_ was highest with RV pre-excitation and decreased up to 33% with LV pre-excitation. These opposite patterns of changes in RV and LV dP/dt_max_ were reproduced by the simulations. The simulations extended these observations by showing that changes in steady-state biventricular cardiac output differed from changes in both LV and RV dP/dt_max_. The model allowed to explain the discrepant changes in dP/dt_max_ and cardiac output by coupling between atria and ventricles as well as between the ventricles.

**Conclusion:** The LV and the RV respond in a opposite manner to variation in the amount of LV or RV pre-excitation. Computer simulations capture LV and RV behavior during pacing delay variation and may be used in the design of new CRT optimization studies.

## Introduction

Cardiac resynchronization therapy (CRT) is an established therapy for heart failure patients with a reduced left ventricular (LV) ejection fraction (EF), and left bundle branch block (LBBB) (Brignole et al., 2013). Through biventricular pacing, CRT aims to establish a more synchronous electrical activation of the ventricles and thereby improves cardiac pump function (Vernooy et al., 2007) and clinical outcome (Brignole et al., 2013). However, approximately one-third of the patients that receive CRT do not benefit from this therapy (Abraham et al., 2002; Cleland et al., 2005; Auricchio and Prinzen, 2011; Prinzen et al., 2013).

Programming of both atrioventricular (AV) and ventriculoventricular (VV) pacing delays strongly influences the contractile response to CRT, as determined by both ultrasound and maximum rate of LV pressure rise (LV dP/dt_max_) (Bogaard et al., 2012; Auger et al., 2013; Strik et al., 2013b). However, meta-analyses of multiple optimization methods showed that pacing delay optimization fails to provide long-term improvement in clinical outcome (Auger et al., 2013). Suggested reasons for the absence of long-term benefits of such optimization are that the default “out-of-the-box” delays are already fairly good and that the optimization methods employed are not accurate or robust enough. An alternative explanation could be that most pacing delay optimization strategies that have been developed solely take LV function into account. Right ventricular (RV) function is often overlooked, although several studies show that RV failure is an independent predictor of mortality in patients with LV failure with and without CRT (Groote et al., 1998; Ricci et al., 2017). Two clinical studies indicated that there was a poor correlation between the pacing delay settings providing the highest LV dP/dt_max_ and RV dP/dt_max_ values (Sciaraffia et al., 2009; Hyde et al., 2016).

In recent years, computational models of cardiac electrophysiology, mechanics of the heart and cardiovascular system have increased our understanding of dyssynchronous heart failure and its treatment with CRT (Lee et al., 2018). Right ventricular function and its effect on CRT has, however, not been studied extensively using a computer modeling approach. Our group has been using the CircAdapt model of the heart and closed-loop circulatory system. While using a simplified cardiac anatomy, the advantages of this model are the inclusion of the entire (systemic and pulmonary) circulation and its high calculation speed (almost real time). In combination with experimental and clinical measurements, CircAdapt has shown to be able to identify and mechanistically understand the electromechanical substrates of the heart that are most responsive to CRT (Lumens et al., 2013, 2015; Huntjens et al., 2018).

A vast majority of the studies on the heart, also our aforementioned studies, are related largely to the LV. In the present study, we aim to study the changes in both LV and RV contractile response to variations of pacing delay settings in CRT, and to evaluate whether the CircAdapt computer model reliably simulates LV and RV pump function during these interventions. Subsequently we aim to use the computer modeling results to investigate how cardiac output is affected by differences between LV and RV contractile changes.

## Materials and Methods

### Animal Experiments

Animal handling was performed according to the Dutch Law on Animal Experimentation and the European Directive for the Protection of Vertebrate Animals Used for Experimental and Other Scientific Purpose. The protocol was approved by the Animal Experimental Committee of Maastricht University. The animal experiments have been conducted in 2010–2011 and the methodology used has partially been described in a previous publication (Strik et al., 2013b), reporting on the LV pressure data. In the current study we analyzed both RV and LV pressure data and related them to the electrical conduction measurements. In short, 6 adult mongrel dogs were anesthetized using midazolam (0,25 mg/kg/h) and sufentanil (3 μg/kg/h) and a complete AV-block was induced by radiofrequency ablation. The dogs received pacing electrodes in the right atrium (A), RV apex and epicardially on the basal posterolateral wall via a left-sided thoracotomy.

**FIGURE 1:**
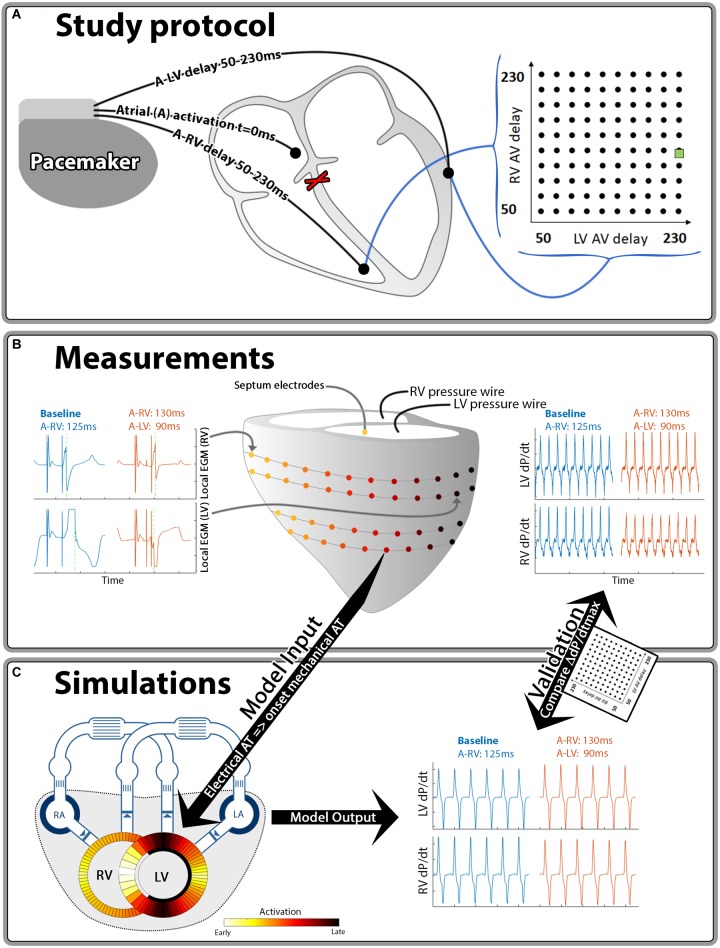
Schematic representation of the methods used in this study. Hundred different A-LV/A-RV pacing delay combinations were programmed **(A)** while pressures and local electrical activation were measured **(B)**. Generic activation maps, derived from local electrograms, were used as onset of mechanical activation in the computer simulations **(C)**. The resulting output of the simulations and measurements was compared for validation purposes. The green square in the heat maps indicates the baseline pacing setting.

Measurements were performed 12–21 weeks after inducing the AV-block using an external custom-built pacing system. RV-only pacing with an A to RV (A-RV) pacing delay of 125 ms was used as the baseline pacing setting, mimicking the activation pattern as seen in LBBB. The A-RV and A to LV (A-LV) delays were then programmed individually, ranging from 50 to 230 ms in steps of 20 ms, resulting in 100 possible combinations (Figure 1A). Pacing delay settings were assigned in a random order and baseline recordings were repeated after every 5th setting. During each setting, continuous, invasive hemodynamic and electrocardiographic measurements were performed (Figure 1B). 7F catheter-tip manometers were used for LV and RV pressure measurement. Epicardial electrograms of the LV free wall (LVFW) and RV free wall (RVFW) were recorded from 2 multielectrode custom-made bands holding 102 contact electrodes. Septal endocardial electrograms were recorded from two multielectrode catheters with 7 contact electrodes on the RV septum and 3 on the LV septum. Measurements were recorded for a minimum of two respiratory cycles at each pacing delay setting.

### Data Analysis

Local electrical activation times were determined as the duration between the atrial pace and the timepoint of steepest negative deflection of the electrogram using custom software (Figure 1B, green line in local EGM’s). For quantification of intraventricular electrical dyssynchrony we calculated total activation time as the difference between earliest and latest activation time of the RVFW (RV TAT) and of the whole LV (LV TAT). The latter was calculated from the combination of epicardial LVFW and endocardial septal electrodes. To quantify electrical interventricular dyssynchrony we used the ventricular electrical uncoupling (VEU) index. VEU was defined as the difference between mean LVFW and mean RVFW activation times (Ploux et al., 2013).

For both the LV and the RV, response to pacing was defined as the relative change in dP/dt_max_ compared to the baseline setting. We applied quadratic LOESS fitting to account for measurement variability within each dog (Cleveland, 1979). Furthermore, in order to quantify a generic canine response to changes in pacing delay, we created a single representative canine dataset by taking the mean values of the dogs for each setting. We also applied linear 2D interpolation between the measurements in the heat map visualizations of all pacing delay settings. All these calculations were performed in Matlab 2016A (The Mathworks Inc., Natick, MA, United States).

### Computer Simulations

The CircAdapt model of the entire heart and circulation (Arts et al., 2005; Lumens et al., 2009; Walmsley et al., 2015a), which can be downloaded from www.circadapt.org, was used to simulate cardiovascular mechanics and hemodynamics during pacing delay variations as applied in the animal experiments (Figure 1C). Previous experimental and clinical studies have shown that the CircAdapt model realistically relates local ventricular myofiber mechanics to global cardiovascular hemodynamics in dyssynchronous and paced hearts (Leenders et al., 2012; Huntjens et al., 2014; Lumens et al., 2015; Walmsley et al., 2015a,b).

The source code of the CircAdapt model used for all simulations as well as the Matlab^®^ (The MathWorks, Natick, MA, United States) scripts to perform all simulations and analysis thereof are provided as an online supplement (Supplementary Data Sheet S1). In summary, the CircAdapt model is a reduced order model of the human four-chamber heart connected to a closed-loop cardiovascular system, with lumped pulmonary and systemic circulations. It uses a simplified ventricular geometry, where cardiac walls are represented by thick spherical shells consisting of contractile myocardium. The MultiPatch module enables cardiac walls to be subdivided into an arbitrary number of wall segments (patches). Tissue properties and activation time can differ between patches, but all patches within a wall share a common wall tension and curvature. Since wall tension is the same in all patches within a wall, spatial location within a wall is not required to calculate deformation in a patch.

In CircAdapt wall tension and curvature determine cavity pressure through Laplace’s law (Lumens et al., 2009; Walmsley et al., 2015a) Fiber stress in a patch is the sum of an active component, representing myofiber contraction, and a passive component. The active stress component incorporates length-dependence of the force generated and the duration of contraction. The passive component provides a non-linear relationship between myofiber stress and strain. More details on the phenomenological model of myocardial contraction and the validation of the MultiPatch module are previously published by Walmsley et al. (2015a).

### Simulating a Healthy Human Reference Heart and Circulation

Cardiac adaptation implemented in the CircAdapt model was used to obtain a reference parameterization that represents a healthy human cardiovascular system (Arts et al., 1994, 2005, 2012). The tissue volumes and areas in the cardiac walls and large blood vessels were adapted as described previously (Arts et al., 2005, 2012). A resting cardiac output of 5.1 l/min and heart rate of 70 bpm were assumed. Cardiac output was tripled and the heart rate was doubled during the stress-state of the adaptation process. Mean arterial pressure (MAP) was maintained at 92 mmHg during the adaptation process. The resulting reference simulation was used as the basis for subsequent pacing simulations.

### Using Electrical Activation to Simulate the Pacing Delay Optimization Protocol

We divided the ventricular wall in the same amount of segments as the number of available electrodes (52 LV free wall, 50 RV free wall and 10 septal segments). Time of onset of activation was assigned based on the electrical activation times measured in the animal experiments. As previously stated, in the current MultiPatch module the segments were considered to be mechanically coupled in series, meaning that the order in which patches were placed was not significant (Walmsley et al., 2015a). This allowed sorting of the activation times per wall in each measurement, before taking the median of the dogs, to get a generic activation pattern. The benefit of this generic activation pattern is that it was less affected by differences in band placement, heart size and electrodes with insufficient contact in the dogs.

A representative baseline simulation was obtained by imposing the ventricular activation pattern measured during the experimental baseline condition, i.e., RV-only pacing with an A-RV delay of 125 ms. Systemic vascular resistance was adapted to obtain a MAP of 60 mmHg and heart rate was set to 80 bpm, both similar to the animal experiments. Furthermore, total circulating blood volume was adjusted so that cardiac output was maintained at 5.1 L/min. The resulting baseline simulation was used as the starting point for the pacing setting simulations. For each of the 100 pacing delay simulations, the pattern of ventricular activation was changed to the activation pattern measured in the canine experiments and the resulting beat-to-beat changes in ventricular mechanics and hemodynamics were stored until a new hemodynamic steady state was reached. During all pacing simulations, systemic vascular resistance and total circulating blood volume were kept constant in order to quantify the acute effect of pacing-induced changes of ventricular pump mechanics and cardiac hemodynamics. Simulated steady-state dP/dt_max_ values are compared with the experimental measurements. In addition, the simulations extended the animal experiments by providing quantitative insight in the beat-to-beat and steady-state changes of ventricular volumes and cardiac output.

## Results

Baseline characteristics for the AV-blocked dogs are described in Table 1.

**Table 1:** Baseline characteristics in median (range) of dogs during baseline (RV-only, A-RV 125 ms pacing).

	During baseline RV-only pacing
Weight (kg)	19.8 (19.4–21.4)
MAP (mmHg)	55 (42–71)
Systolic arterial pressure (mmHg)	70 (62–81)
Diastolic arterial pressure (mmHg)	45 (31–53)
LV dP/dt_max_ (mmHg/s)	1205 (1183–1646)
RV dP/dt_max_ (mmHg/s)	520 (345–700)
Weeks between AVB and Sacrifice (weeks)	13 (12–21)

### Electrical Effects of Altering Pacing Delay Settings

**FIGURE 2:**
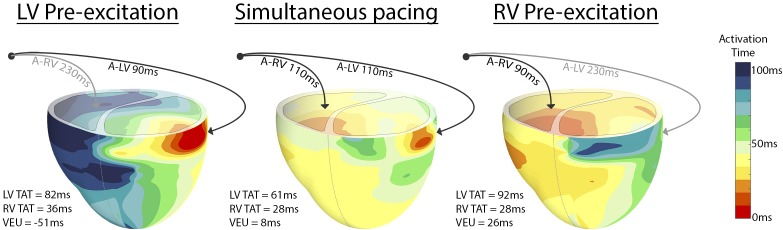
Epicardial electric activation maps in a paced dog heart with complete AV block during LV pre-excitation **(left)**, simultaneous pacing **(middle)**, and RV pre-excitation **(right)**. Black arrows indicate capture, whereas gray arrows indicate loss of capture.

Figure 2 shows the typical examples of electrical activation patterns acquired using contact mapping in a dog with AV block during LV pre-excitation, simultaneous RV and LV pacing and RV pre-excitation. In case of extreme pre-excitation, capture in the last paced ventricle was lost due to activation via the contralateral ventricle (indicated by the gray line). RV pre-excitation led to the largest LV TAT while LV pre-excitation resulted in an increase of RV TAT. During simultaneous pacing, two wave fronts originating from the RV and LV pacing electrodes fused and resynchronized the heart as indicated by a decrease in LV TAT.

**FIGURE 3:**
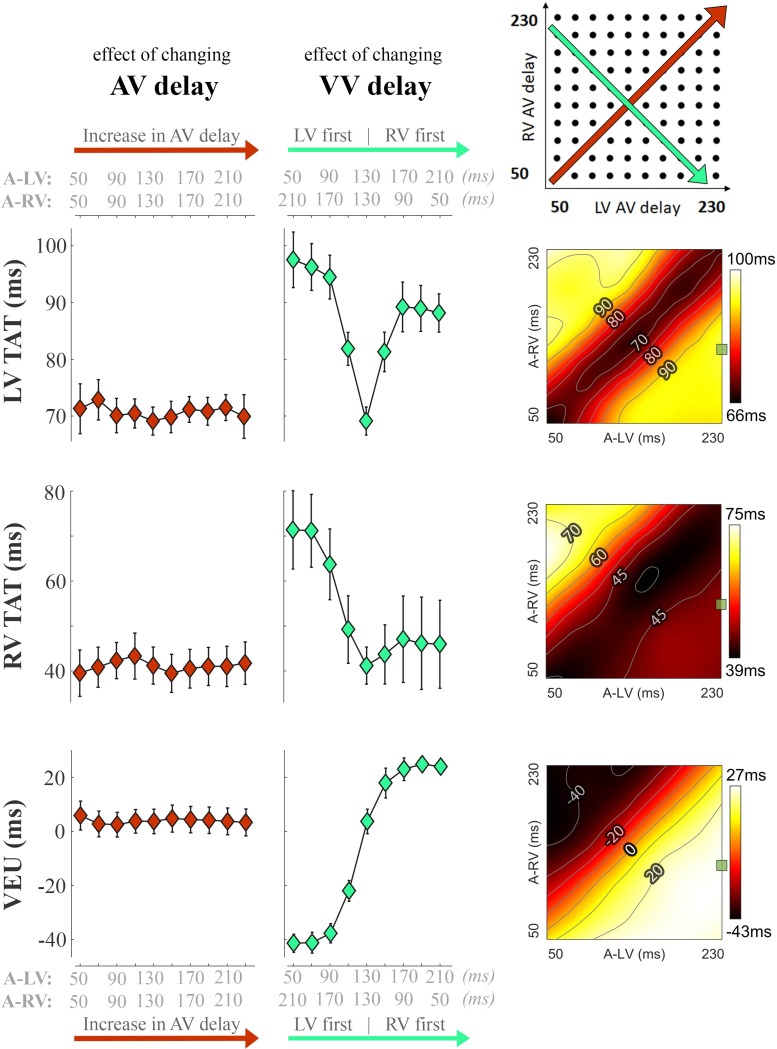
Changes in electrical dyssynchrony indices during variation in pacing delay in the animal experiments. The left column shows the effect of increasing AV delay during simultaneous RV + LV pacing; the middle column shows changes in VV delay (green, from LV pre-excitation to RV pre-excitation) and the heat maps on the right are the results for all pacing setting (mean of six dogs, bars represent standard errors of the mean). From top to bottom: Total activation time (TAT) of the total LV (free wall and septum), RV free wall (RVFW), and VEU (Ventricular Electrical Uncoupling). The green square in each heat map indicate the baseline pacing setting.

Figure 3 shows the changes in electrical dyssynchrony indexes in dogs with variation of pacing delay settings. There was no change in LV and RV TAT and VEU when changing the AV delay during simultaneous activation of the LV and RV (left column). Both LV and RV TAT (first 2 rows of Figure 3) were lowest during simultaneous RV + LV pacing. The RV showed the largest TAT during LV pre-excitation or LV-only pacing (upper left corners in the heat maps). LV TAT showed a relatively large increase with large RV pre-excitation (right side of middle panels and lower right corners in heat maps), while RV TAT did not increase much. As indicated by the VEU (bottom row Figure 3), during LV-only pacing the LVFW was activated more than 40 ms before the RVFW. During RV-only pacing the LVFW was, on average, activated more than 20 ms later than the RVFW. The LVFW and RVFW were activated simultaneously with very slight LV pre-excitation (Figure 3 bottom right, VEU = 0).

### Hemodynamic Effects of Altering Pacing Delay Settings

**FIGURE 4:**
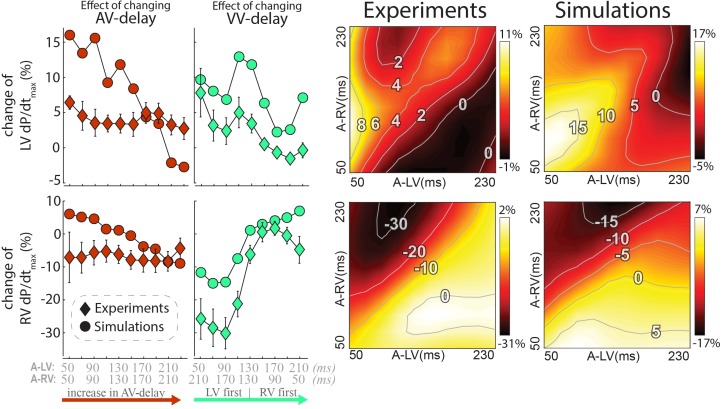
Changes in contractile response as a result of changes in pacing delays in experiments and simulations. Percentile change from baseline of LV dP/dt_max_ (top) and RV dP/dt_max_ (bottom). The left rows depict the same AV and VV delay settings as in Figure 3 are shown. Heat maps for both the experiment (3rd column) and simulations (4th column). Diamonds: Canine measurements (Mean (standard error of the mean) of six dogs; Circles: Simulation output.

The changes in LV and RV dP/dt_max_ in response to changes in pacing delay settings are presented in Figure 4 (top and bottom, respectively). Increasing AV delay during simultaneous RV and LV pacing (left column and identity line in the heat maps) hardly affected LV and RV dP/dt_max_ in both measurements and simulations. The relative effect of changing VV delay (green, second column from left) was largest in RV dP/dt_max_. Changing pacing settings from RV to LV pre-excitation decreased RV dP/dt_max_ with more than 30% in the experiment. The decrease in RV dP/dt_max_ was less pronounced in the simulations but followed the same pattern. LV dP/dt_max_ was highest with LV pre-excitation and simultaneous pacing in both the experiment and simulations.

The heat maps of both the animal experiments and computer simulations (right side of Figure 4) show a qualitatively similar pattern where the largest changes in both LV and RV dP/dt_max_ are observed when changing the VV delay. The largest increase in LV dP/dt_max_ in the measurements was reached with a short A-LV (50 ms) and A-RV (90 ms). LV pre-excitation led to a larger increase in LV dP/dt_max_ than RV pre-excitation during all measurements and simulation, with an optimal LV pre-excitation range of 10–50 ms. For RV dP/dt_max_, all LV pre-excitation pacing settings led to a decrease up to 33% in the experiment and 18% in the simulations, while RV pre-excitation caused little change compared to baseline (RV-only) pacing.

### Changes in Simulated Cardiac Output at Different Pacing Delays

**FIGURE 5:**
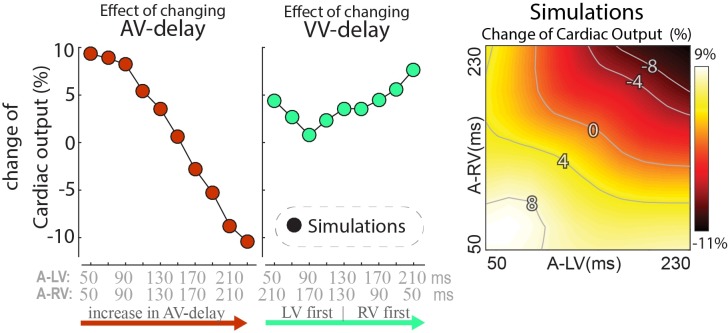
Relative change in simulated steady-state cardiac output with a change in pacing delay settings. Depicted are the changes relative to baseline (see text).

While dP/dt_max_ values are regarded as a measure of ventricular contractility, cardiac output may be more closely related to pump function of the entire heart. Note that due to the closed loop circulation, in a steady state situation cardiac output of the RV and LV are the same. Cardiac output was not determined in the experiments, but it was calculated in the model simulations. In these simulations the changes in cardiac output following a switch in pacing delay differed from the changes in both LV and RV dP/dt_max_ (Figure 5). Cardiac output was more sensitive to changes in AV-delay than to changes in VV-delay. AV-delays of 50 and 70ms led to the largest increases in cardiac output, amounting up to 9%.

**FIGURE 6:**
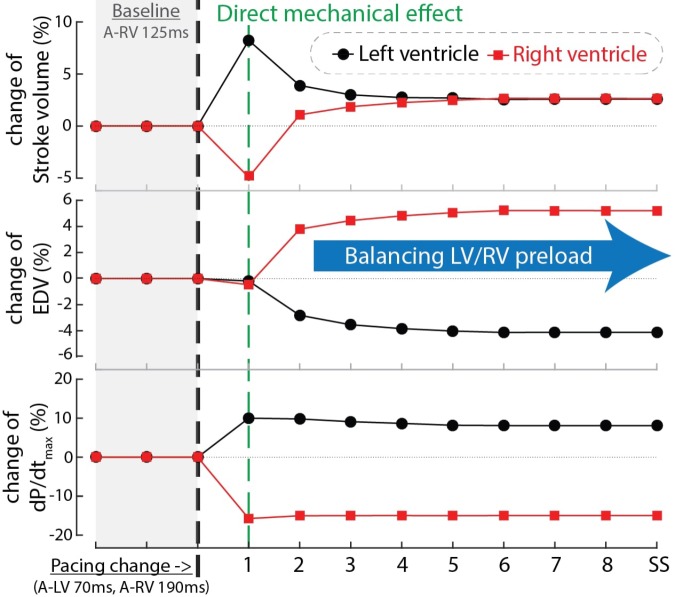
Time courses of the relative change of stroke volume (top), end-diastolic volume (EDV, mid) and dP/dt_max_ of the LV (black circles) and RV (red squares) after changing pacing delay from baseline (A-RV 125 ms) to LV pre-excitation (A-LV 70 ms, A-RV 190 ms) in computer simulations. The black dashed line indicates the start of the change in pacing delay. The numbers in the blue bar indicate the number of simulated cardiac cycles. SS, steady state.

In order to find an explanation for the differences in behavior between cardiac output and RV and LV dP/dt_max_ we compared the time course of these parameters as well as EDV during the first beats after start of a certain setting, in this case LV pre-excitation (Figure 6). In the first beat after the change in pacing setting and therefore also activation sequence (see above), both LV stroke volume and dP/dt_max_ increased while RV stroke volume and dP/dt_max_ decreased. In the subsequent beat RV EDV increased, due to the smaller SV of the previous beat, whereas LVEDV decreased. As a consequence of these EDV changes, RV SV recovered and LV SV decreased to some extent and in the third and subsequent beats an steady state (SS) was reached, with SV in both ventricles (and therefore cardiac output) increasing by about 3%. This example, representative for the other conditions, illustrates that dP/dt_max_ was largely independent of preload, whereas SV depended on it, most likely due to the length dependent activation, implemented in the CircAdapt model (see section “Materials and Methods”).

**FIGURE 7:**
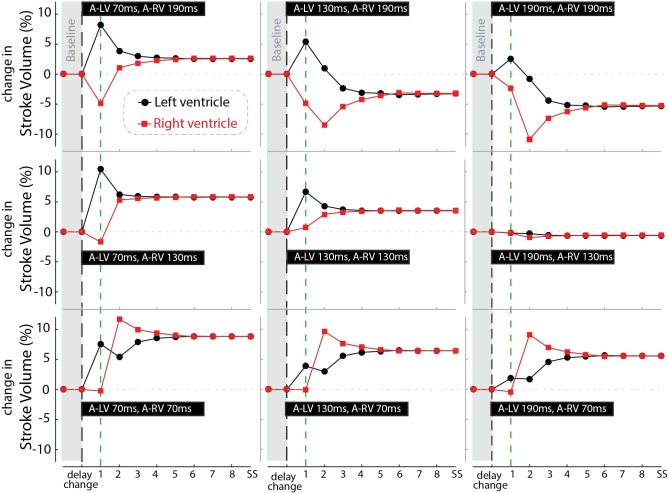
Relative change in stroke volume of the left (black circles) and right (red squares) ventricle over a number of simulated cycles until steady-state (SS) after changing pacing delay (the nine settings shown in the black bars). Black dashed line indicates the moment of changing the pacing delays while the green line marks the first beat.

Figure 7 shows the response of stroke volume of the LV and RV after simulated programming of nine different pacing settings. In the first beat RV SV remained either unchanged or decreased as compared to baseline, indicating little direct mechanical benefit of the change in pacing delay for the RV. However, similar to the example in Figure 6, copied into the left upper panel of Figure 7, RV stroke volume changed considerably in subsequent beats. While changes in LV SV initially differed from RV SV, a steady state was reached after several simulated beats. Note that the largest benefit in SV, and therefore cardiac output, was primarily dependent on AV-delay. For example, in the bottom row (A-RV 70 ms) the optimized RV filling improved stroke volume to such a degree that, through the serial coupling, LV preload increased, leading to a further increase in LV stroke volume after the second cycle. On the other hand, at longer AV-delays this atrial-ventricular coupling decreases, resulting in a lower steady-state cardiac output.

## Discussion

In this study we investigated the influence of LV and RV pacing delay settings on LV and RV electrical activation and contractility in animal studies and computer simulations. Both studies showed that LV TAT is smallest during synchronous RV and LV stimulation and increases when VV delays increase. RV TAT becomes larger in particular during LV pre-excitation. LV and RV contractility vary most, and in opposite direction, with changes in VV delay settings. After demonstrating the realistic simulations in the model, we used the model to calculate cardiac output changes and to explain why changes cardiac output differed from both RV and LV contractility. The latter findings demonstrate how a model like CircAdapt can extend mechanistic understanding of circulatory changes due to a device therapy.

### LV and RV Contractility Respond in Opposite Manner to Variations in VV Pacing Delays

A key finding in the present study is that LV and RV dP/dt_max_ change in opposite direction when changing the VV pacing delay. Sciaraffia et al. (2009) previously demonstrated that RV and LV dP/dt_max_ identify different “optimal” VV delays in most of the patients included in their study. Furthermore, Houston et al. (2018) recently showed that in patients with dyssynchronous heart failure, RV-only pacing leads to higher RV dP/dt_max_ than LV-only or simultaneous LV + RV pacing. In an experimental study in pigs, different VV delays were tested at different pacing locations (Quinn et al., 2006). Similar to our study, these investigators found that RV pre-excitation led to a higher RV dP/dt_max_ than LV pre-excitation. The fact that findings were consistent in patients, animals and a computer model implies that the opposing changes in hemodynamics, caused by varying VV pacing delays, are caused by a universal mechanism.

Another key finding is that ventricular specific pre-excitation is required for a good contractile function in both the LV and RV. This is illustrated by LV pre-excitation increasing LV dP/dt_max_ and RV pre-excitation leading to the largest RV dP/dt_max_ values. In contrast, changes in AV delay have less effect on measured and simulated LV and RV dP/dt_max_. In a previous study, in which we evaluated the relative importance of interventricular and intraventricular dyssynchrony for contractile response to CRT (change in LV dP/dt_max_), it was demonstrated that interventricular dyssynchrony during intrinsic rhythm is the dominant electrical substrate driving response to CRT (Huntjens et al., 2018). In contrast, intraventricular dyssynchrony showed little effect on LV dP/dt_max_, which is in line with experimental observations that reducing LV TAT by multipoint pacing does not improve LV dP/dt_max_ (Ploux et al., 2014). However, results in this study suggest that intraventricular dyssynchrony might still play a modulating role since increase in LV TAT, with large LV pre-excitation, led to a decrease in LV dP/dt_max_ compared to slight LV pre-excitation. Because RV TAT increases concurrently with decreases in VEU we cannot distinguish if either intra- or interventricular dyssynchrony has a larger effect on RV contractility.

In agreement with our observations, other studies in patients have shown that the lowest electrical dyssynchrony does not necessarily lead to the highest LV contractility or better clinical outcome (Thibault et al., 2011; Lumens et al., 2013). Optimization based on minimization of electrical dyssynchrony alone might therefore not be sufficient. Even if the optimal electrical activation for LV contractility is known, further clinical studies are required to investigate whether the gain in LV contractility outweighs the loss of RV contractility, especially since cardiac output might not match either or both but be a combination/compromise.

### CircAdapt Simulations Capture Both LV and RV Contractile Response to Pacing Delay Changes

The present study demonstrates that the CircAdapt model can capture pacing-induced changes to both LV and RV contractile function. It is especially the response of the RV to pacing that has been less well studied, both by our group and by others.

The use of experimental measurements electrical activation of the paced dog heart, derived from canine experiments, coupled to the simulation of the entire circulation, resulted in changes in RV and LV dP/dt_max_ that closely mimicked values measured in dog experiments.

Earlier studies have shown that CircAdapt enables realistic simulation of cardiac response to CRT, mostly focusing on LV function (Lumens et al., 2013, 2015; Huntjens et al., 2018). In one of those studies, it was demonstrated that the RV plays an important role in the improvement of LV function during LV-only pacing (Lumens et al., 2013). The present study extends the mechanistic insight in the working action of CRT in the context of pacing delay optimization, where the complex mechanical and hemodynamic interactions between the four cardiac cavities and the surrounding circulations are found to be important.

As demonstrated in this study, the CircAdapt model can capture the complexity of different LV and RV responses to CRT by incorporating several relevant components of cardiovascular interaction. Firstly, it realistically incorporates direct mechanical interaction between the three ventricular walls (Lumens et al., 2009). Secondly, it allows realistic simulation of regional myocardial mechanics in the ventricular walls of the asynchronously activated heart (Leenders et al., 2012; Walmsley et al., 2015a), thereby enabling experimentally measured activation times to be imposed and the related intraventricular heterogeneities in mechanical myofiber behavior to be simulated. Thirdly, it is a closed-loop system allowing for indirect (serial) hemodynamic interaction between the left and right side of the heart through the systemic and pulmonary circulations (Arts et al., 2005; Lumens et al., 2010). Fourthly, its four-chamber heart captures the dynamics of hemodynamic atrioventricular interactions (Jones et al., 2017). Lastly, it includes the mechanical interaction through pericardial constraint, with an increase in the volume of a chamber altering the pressure in the other chambers and, hence, diastolic filling and septal position (Palau-Caballero et al., 2017).

### Simulation-Derived Mechanistic Insights

The ability of CircAdapt to realistically simulate both LV and RV hemodynamics during pacing allowed us to further study the impact of differences between both chambers on cardiac output. In particular, the model showed a difference in response between dP/dt_max_ and SV/CO. Similar differences have been observed in a clinical study where LV dP/dt_max_ responses differed from responses in stroke work (van Everdingen et al., 2018).

Our simulations provided a plausible explanation for this paradoxical observation. While LV and RV stroke volume of the first beat after a change of pacing delay can differ substantially, a common steady-state stroke volume and thereby cardiac output is reached due to balancing ventricular preload conditions during the next few beats. There are three main determinants of the newly achieved steady-state preload condition. First, the changes in stroke volumes directly change the end-systolic volumes and thereby the end-diastolic volumes in the next beat. Second, the pacing-induced changes in effective left and right AV delays change the efficiency of atrial and ventricular diastolic filling and subsequent systolic contraction. Thirdly, changes in stroke volume affect the filling of the other ventricle through the systemic and pulmonary circulations.

While dP/dt_max_ changes congruently with first-beat stroke volume in the same ventricle, it is less affected by changes in preload. As a result, LV and RV dP/dt_max_ are much more sensitive to changes of VV-delay and, hence, asynchrony of electrical activation than to changes of AV-delay. On the other hand, changes of AV-delay affect cardiac output more than LV and RV dP/dt_max_.

### Computer Modeling in Therapy Optimization

Other cardiac computer modeling studies have been conducted to investigate other factors in the optimization of CRT therapy. For example, in a cohort of 648 virtual patients it was found that the location of the LV pacing site is an important factor in response to CRT (Crozier et al., 2016). Electrophysical cardiac computer modeling studies also demonstrated the importance of LV pacing site and the potential of simulations to predict the electrical optimal pacing location and setting (Reumann et al., 2007; Miri et al., 2009; Niederer et al., 2011). Our study shows, however, that an electrical optimum (lowest electrical dyssynchrony) might not necessarily be optimal for overall pump function.

The CircAdapt simulations performed for this study can run on a single core in real time. CircAdapt requires activation time as input and lacks the cardiac electrophysiological model necessary to extract this information from standard clinical data. This input could, however, potentially be generated by other models, for example the ones referred to in the previous paragraph. This would also allow for testing of alternative pacing sites, which would result in different activation patterns, which subsequently can be used as input for CircAdapt simulations. A workflow where fast and anatomically realistic cardiac electrophysiological simulations are combined with cardiac mechanical and circulatory CircAdapt simulations might further increase the clinical applicability of cardiac computer models.

### Study Limitations

In this study the pacing experiments were performed in relatively healthy canine hearts. Previous work from our group showed that chronic total AV-block leads to structural changes (hypertrophy) and electrical remodeling (QT-time prolongation), but normal contractility (Peschar et al., 2003). On the other hand, patients treated with CRT have different levels of myocardial remodeling and heart failure, which may affect the response to pacing (Strik et al., 2013a, Nguyên et al., 2018). Results of this study might differ from patient data since long-term structural remodeling was not included in both the animal and computational experiments. On the other hand, differences in RV and LV response observed in this study could mean that different pacing settings might affect the positive and/or negative remodeling of the LV and RV.

A major difference between this animal experiment and patients in day-to-day life is that the animals were anesthetized. To allow comparison between the experiments and simulations, the model’s LV afterload in the baseline situation was adapted to fit the measured mean arterial pressure in the dogs. After the change in activation delays regulation was disabled, which is likely similar to the anesthetic condition were regulation is slow. Caution should, however, be taken when translating results of this study toward the clinical setting considering that loading conditions potentially affect the effect of pacing delay changes (Quinn et al., 2009). Future studies are needed to investigate how load-dependent the observed effects of pacing delay optimization are and how homeostatic regulation interacts with changes in LV and RV contractility. A final limitation of the CircAdapt (and most other computer models in this field) is that changes in function of the autonomic nervous system are not taken into account.

### Clinical Perspective

The results of this study raise the question what outcome measure is best to use for optimization of pacing delay. Current clinical practice focusses almost exclusively on the LV, using parameters like LV dP/dt_max_, aortic outflow integral, and LV systolic (or aortic) pressure. This study demonstrates that improving LV function can reduce RV function. Furthermore, cardiac output is not necessarily increasing when LV dP/dt_max_ increases. Hence, An exclusive focus on the LV might not lead to the best overall outcome. Therefore, future studies on optimization of therapy should not exclusively focus on the LV but also include measures of RV and/or whole heart function. In our analysis of cardiac output we also demonstrated that the moment of measurement affects what physiological phenomenon is actually observed. Contractile function alone might better be observed in the first beats while longer lasting measurements, that allow reaching a steady state, will provide more information about the loading of the heart and its interaction with homeostatic regulation. Insights acquired in the present opens the way for designing better optimization protocols, possibly even including computer modeling.

## Conclusion

The LV and the RV respond in an opposite manner to LV or RV pre-excitation. LV pre-excitation improved LV contractility and decreased RV contractility, while RV pre-excitation had the opposite effects. The CircAdapt computer model realistically captures these opposite responses of LV and RV contractile function. Computer simulations extend animal experimental findings by revealing that improving ventricular contractility does not necessarily lead to an improvement of cardiac output. This study demonstrates the potential of CircAdapt to provide a valuable and efficient *in silico* platform for further optimization studies for device therapy.

## Author Contributions

EW, PH, MS, JW, FP, and JL conceived and designed the experiments. EW, RS, and MS performed the experiments. EW, RS, PH, MS, GP, EV, FP, and JL analyzed and interpreted the data. MS, JW, FP, and JL contributed to materials and analysis tools. EW, RS, PH, MS, GP, EV, JW, KV, TD, FP, and JL wrote or provided the critical revision on the manuscript.

## Conflict of Interest Statement

The authors declare that the research was conducted in the absence of any commercial or financial relationships that could be construed as a potential conflict of interest.
